# Maternal HIV Stigma and Child Adjustment: Qualitative and Quantitative Perspectives

**DOI:** 10.1007/s10826-021-02034-x

**Published:** 2021-07-10

**Authors:** Sae-Jin Kim, Abigail Robbertz, Nada M. Goodrum, Lisa P. Armistead, Lindsey L. Cohen, Marya T. Schulte, Debra A. Murphy

**Affiliations:** 1 Department of Psychology, Georgia State University, Atlanta, GA 30303-5010, USA; 2 Department of Psychology, University of South Carolina, 1512 Pendleton Street, Barnwell College, Suite #220, Columbia, SC 29208, USA; 3 Department of Psychiatry, University of California Los Angeles, 757 Westwood Plaza, Los Angeles, CA 90095, USA

**Keywords:** HIV, stigma, child adjustment, parent-child relationship, mixed methods

## Abstract

Globally, over 36 million people are living with HIV, and 51% are women, most of whom are of childbearing age. Children of mothers with HIV exhibit more adjustment problems than their demographically similar peers. Using mixed methods, this study examined associations between mothers’ HIV stigma and their children’s functioning. As part of a multi-site study in Georgia and California, participants included 181 HIV-positive mothers and one of their 6- to 14-year-old children. Mothers reported on children’s psychological adjustment, their own anxiety, and their perceived HIV stigma. Children reported on parent-child relationship quality. Following the quantitative portion of the study, 14 mothers and 13 children who knew their mothers were HIV positive completed in-depth qualitative interviews addressing the impact of HIV disclosure on family relationships. Hierarchical regression analyses found that parent-child relationship quality, maternal anxiety, and HIV stigma were significantly associated with child adjustment difficulties. HIV stigma predicted child adjustment problems after accounting for the role of parent-child relationship and maternal anxiety. Most mothers and some children in the qualitative portion of the study discussed the impact HIV stigma on their lives. In all, despite efforts to decrease HIV stigma, it continues to affect the lives of those with the virus and their families. Interventions, then, should not only focus on the parent-child relationship and maternal anxiety when attending to child functioning in families affected by HIV, but should also target coping skills for mitigating the impact of stigma while continuing efforts to reduce HIV-related stigma.

## Introduction

Despite considerable advances in treatment, human immunodeficiency virus (HIV) remains a significant public health concern of global proportions. There are more than 38 million people living with HIV worldwide with 1.7 million people testing HIV positive (+) in 2019 alone (World Health Organization [WHO], 2021). Women account for approximately 51% of the population of people living with HIV (PLH). Women tend to be the primary caregivers for children (WHO, 2018), and with the development and widespread implementation of anti-retroviral therapy (ART), HIV + women are increasingly able to live longer and raise their children into adulthood (Chi & Li, 2013; Samji et al., 2013). Thus, there are important implications in studying the psychosocial adjustment of children and adolescents of mothers living with HIV (MLH).

Researchers have consistently found significant associations between mother-child relationship quality, as well as maternal anxiety, on child psychosocial adjustment in HIV-affected families with women as the primary caregivers (Forehand et al., 2002; Murphy et al., 2010). A handful of studies have examined MLH stigma and its connections to child psychosocial functioning (Bauman et al., 2002; Murphy et al., 2002; Murphy et al., 2006). Given the centrality of stigma to the experience of families affected by maternal HIV and data indicating that stigma is related to health indicators such as viral suppression (Lipira et al., 2018), additional study of stigma in MLH families is war-ranted (Baugher et al., 2017; Gamarel et al., 2017). In this study, we employed mixed methods to examine mothers’ HIV-related stigma and whether it was related to their children’s adjustment above and beyond two factors consistently demonstrated to impact child functioning in HIV-affected families: parent-child relationship quality and maternal anxiety.

### Parent-Child Relationship Quality and Child Adjustment

Parent-child relationship quality refers to each individual’s affective appraisal of the other person’s behaviors in the dyad, as well as their interactions with one another (Prinz et al., 1979). Child adjustment, on the other hand, is the child’s ability to deal with stressful situations in the environment through inward (e.g., positive or negative self-statements) or outward (e.g., seeking or avoiding social support) processes. This conceptualization for psychosocial adjustment is consistent with how psychological disorders or lower levels of adjustment broadly map onto internalizing (e.g., depression) or externalizing (e.g., aggression) spectrums in metanalytic study (Achenbach, 1991; Krueger & Markon, 2006).

Overall, researchers have demonstrated a significant positive association between parent-child relationship quality and child adjustment in a variety of populations, including MLH and their children (Bauman et al., 2002; Chi & Li, 2013; Forehand et al., 2002). Notably, Forehand et al. (2002) found that MLH-child relationship quality was longitudinally associated with adjustment difficulties in children, such that improvement in relationship quality over time, led to children being less likely to experience adjustment difficulties at the final data collection point. This finding strengthens the evidence for MLH-child relationship as a protective factor for child adjustment. The researchers also revealed a significant interaction effect such that mother-child relationship quality was less strongly correlated to child adjustment in families affected by maternal HIV than those non-affected. The results from Forehand et al. suggest that even though parent-child relationship quality is a significant protective factor for children’s psychosocial adjustment, alternative risk and protective factors need to be considered in conjunction with parental relationship quality for families affected by maternal HIV as compared to demographically similar families not affected by HIV. For example, maternal anxiety and HIV-related stigma may be particularly relevant predictors of adjustment for children of MLH.

### Maternal Anxiety and Child Adjustment

The negative relationship between parental anxiety and child adjustment is well documented in the literature (e.g., Burstein et al., 2010). In particular, higher levels of parental anxiety are associated with more internalizing problems in children than seen in children with parents exhibiting lower levels of parental anxiety. Research has demonstrated similar yet more nuanced results in families affected by maternal HIV (Hough et al., 2003; Murphy et al., 2010). Murphy et al. (2010) examined the relationship between parental anxiety/stress, parenting skills, and child adjustment with MLH and their children. As reflected in their terminology, the researchers used a broader conceptualization of parental anxiety, which included the construct of parental anxiety used in this current paper in addition to health-related anxiety and parental stress (Abidin, 1983; Achenbach, 1991; Murphy et al., 2001; Veit & Ware, 1983). The study revealed that parental anxiety/stress had an indirect negative association with child adjustment through parenting skills. In other words, parental anxiety/stress experienced by MLH was negatively associated with their parenting skills, and those deficits in parenting skills were likewise negatively associated with child adjustment. Thus, for families affected by maternal HIV, the current research indicates that MLH’s anxiety is a significant risk factor for problems in child adjustment, but the pathway through which maternal anxiety influences child adjustment is nuanced and multifaceted and should be considered in the context of other risk and protective factors.

### Maternal HIV Stigma and Child Adjustment

HIV-related stigma continues to be a primary concern for PLH in the United States (Baugher et al., 2017; Rice et al., 2019) and beyond (Li et al., 2009; Williams et al., 2019). A majority of men and women report prejudiced attitudes towards PLH in approximately 35% of countries where data are available (Joint United Nations Programme on HIV/AIDS [UNAIDS], 2015). Stigma is defined as “an attribute that is deeply discrediting” according to society, which diminishes the stigmatized individual from “a whole and usual person to a tainted, discounted one” (Goffman, 1963, p. 3). Thus, stigma is a socially constructed process, meaning that society determines what is “discrediting” and thereby develops a structure that delineates how the bearers of this stigma and those associated with the individual are devalued across their relationships (Goffman, 1963). For MLH, HIV stigma might include the fear of negative attitudes, judgment, and discrimination based on HIV status and serostatus disclosure (i.e., perceived stigma); the acceptance of negative stereotypes associated with HIV as part of the self or identity (i.e., internalized stigma); and the actual experience of discrimination (i.e., enacted stigma; Berger et al., 2001; Earnshaw & Chaudoir, 2009; Nyblade, 2006).

Most of the research on HIV-related stigma has focused on its association with negative outcomes in the physical and mental health of PLH, including worse adherence to antiretroviral therapy (Earnshaw et al., 2013; Katz et al., 2013; Rice et al., 2019), higher depressive symptoms (Onyebuchi-Iwudibia & Brown, 2014; Rao et al., 2012), and lower social support (Rueda et al., 2016; Sayles et al., 2008). A few studies have examined the potential relation between PLH’s stigma on the adjustment of others within their immediate social context, which is important to consider given that stigma represents a dynamic social process experienced by the stigmatized individual and the people with whom they interact (Parker & Aggleton, 2003). For example, in HIV-affected populations, family members are likely to encounter some level of HIV-related stigma and discrimination even if they themselves are serostatus negative for HIV (Bogart et al., 2008).

Supportive of the conceptualization of HIV stigma as a shared experience, researchers have demonstrated that children’s perception of stigma related to having a HIV + parent is negatively associated with outcomes in their adjustment (Chi & Li, 2013; Chi et al., 2016; Murphy et al., 2002). Similarly, scientists have found a negative association between parents’ perceived HIV stigma and child adjustment in families affected by the epidemic, though the results of this research have been more varied and limited (Bauman et al., 2002; Gamarel et al., 2017; Murphy et al., 2006). Specifically, Bauman et al. (2002) determined that there was no significant relationship between maternal HIV stigma and behavioral problems (i.e., externalizing difficulties) in child adjustment with MLH and their serostatus negative children. In contrast, Gamarel et al. (2017) revealed a positive association between parental stigma and internalizing difficulties in a sample of South African families affected by HIV. Murphy et al. (2006), in contrast, found that maternal HIV stigma was not related to children’s depression (i.e., internalizing dimension) yet was positively associated with children’s delinquent behavior (i.e., externalizing dimension) for both children that were aware and unaware of their mother’s positive serostatus.

Generalizability of the Bauman et al. (2002) and Gamarel et al. (2017) findings are limited. Specifically, Bauman et al. employed a restricted operationalization of child adjustment as only externalizing problems were examined, whereas Gamarel et al. sampled a culturally unique and diverse group of families affected by paternal, maternal, or child HIV. Thus, while there are some indications that maternal HIV stigma is a risk factor for child adjustment, the mixed and limited results of the research highlight a need for further study of relations among these constructs, particularly in the context of the more established protective and risk factors of parent-child relationship quality and maternal anxiety.

### Cognitive-Affective-Behavioral Model of Concealing Stigma

As none of the MLH participants in our sample had disclosed their positive serostatus to their child at the time of quantitative data collection, we adopted a cognitive-behavioral-affective model for the concealment of stigma (Pachankis, 2007). According to Pachankis (2007), concealed stigma holds negative cognitive (e.g., preoccupation and vigilance) and affective (e.g., anxiety and depression) implications for the psychosocial adjustment of the stigmatized individual or MLH. These immediate cognitive and affective complications, in turn, exacerbate each other in a bidirectional manner and subsequently predict increased problems in behavioral functioning (e.g., impression management and impaired close relationship functioning). The *maladaptive behaviors in close relationships* component in the Pachankis model is particularly relevant to the current study given the importance of the MLH as a caregiving figure for the child and the negative indirect relationship between maternal anxiety and child adjustment through parenting skills and parent-child relationship quality (Hough et al., 2003; Murphy et al., 2010). In all, Pachankis’ theory for the concealment of stigma provides a framework for maternal HIV stigma as a distal but significant risk factor for problems in child adjustment via affective and behavioral pathways that map well onto the broader empirical literature.

### Aims and Hypothesis

In this study, we aimed to disambiguate the additive effects of mother-child relationship quality, maternal anxiety, and maternal HIV stigma on child psychosocial adjustment with MLH and their serostatus negative children. While previous research has established mother-child relationship quality as a protective factor (Bauman et al., 2002; Chi & Li, 2013; Forehand et al., 2002) and maternal anxiety as a risk factor for child adjustment (Hough et al., 2003; Murphy et al., 2010) in families affected by maternal HIV, the research on the potential association between maternal HIV stigma and child functioning is incomplete and varied. In consideration of the underdeveloped nature of this literature base, we incorporated qualitative analysis, exploring the thematic content about HIV stigma reported from interviews with MLH and their children. Moreover, a cognitive-behavioral-affective model for the concealment of stigma (Pachankis, 2007) supplemented and informed the quantitative analyses for this study. Based on this theoretical model and previous research (Gamarel et al., 2017; Murphy et al., 2006), we hypothesized that above and beyond mother-child relationship quality and mother anxiety, maternal HIV stigma would negatively predict child psychosocial adjustment in families affected by maternal HIV.

## Method

### Participants

Participants were part of a randomized clinical trial examining the efficacy of an intervention designed to assist MLH with disclosure of their serostatus to children. We used baseline data from this trial for quantitative portion of the current study as reported by 181 MLH and one of their HIV-negative children (50.3% female). At the time of quantitative data collection, children had not been told of their mothers’ HIV + status. Mothers and their children were on average 39 (*SD* = 7.71) and 10 (*SD* = 2.49) years old, respectively. Participants lived in California or Georgia with a primarily Black/African American (56.1% of mothers and 57.2% of children) or White (34.4% of mothers and 31.1% of children) racial background. Around one-third of participants (33% of mothers, 35% of children) identified as Latino or Hispanic. Interviews were conducted in the participant’s preferred language, either English or Spanish. Mothers were primarily single parents (44%), had other adults living in the home (52.6%), completed at least high school (55.5%), and had an average monthly income of $1200.

Participants included in the qualitative interviews were 14 mothers and 13 children. These participants were recruited after the child had been told of their mother’s HIV status, which occurred after the data used in the quantitative portion of this study had been collected. Six families were recruited from the Georgia site, and eight were recruited from the California site. The Georgia families were African American, and the California families identified as Latinx. Six of the children were male, and the average age of all child participants was 12.5 (SD = 1.91). Twelve of these mothers were in the intervention group, and the other two mothers were in the waitlist control condition. These two mothers received the intervention in a group format after completing all quantitative interviews.

### Procedure

University institutional review boards approved this National Institute of Mental Health-funded study to test the efficacy of an intervention called Teaching, Raising, And Communicating with Kids (TRACK), designed to help mothers disclose their HIV status to their children. All study procedures were completed at the mother’s home or a private location selected by the mother. Screener questions were used to assess eligibility criteria followed by consent and assent processes for the mother and child, respectively. Staff were trained in interviewing participants, recruitment, consent, handling adverse events, and tracking/retention efforts.

Participants were recruited through community workshops, social services, ads on public transportation, and medical organizations. Eligibility criteria included being English or Spanish speaking, mother’s confirmed HIV/AIDS diagnosis, being the primary caregiver of an HIV-negative child between the ages of 6–14, the child being unaware of mother’s HIV status (at the time of quantitative baseline data collection) and having no mental health or developmental disorders that could impact their ability to respond to interviewing. If a mother had more than one child that met the inclusion criteria, one of the children was randomly selected for participation.

#### Quantitative data collection

Data were collected at four time points: baseline, 3-, 9-, and 15-months using computer-assisted personal interviewing (CAPI) software. After baseline, participants were randomized to the intervention or control group. Mothers receiving the intervention had three 75-minute sessions with an intervention facilitator to learn information and strategies to help disclose their HIV status to their children. For this study, we only evaluated data from the baseline interview.

#### Qualitative data collection

Purposive sampling (Miles & Huberman, 1994) was used to recruit a sample of mothers and their children who had completed the TRACK study (i.e., participated or passed the point of participation in the 15-month follow-up assessment). Qualitative interviews with these mothers and their children were conducted separately by two graduate research assistants. Mothers consented for the child to be interviewed about their mother’s HIV and the disclosure process, and children assented to be interviewed.

The semi-structured interviews were developed in line with the larger study goals of mothers’ disclosure of their HIV status. The interviews included questions regarding parenting (e.g., “Tell me about how your mother is as a parent.” or “Tell me about what is hardest about being a parent living with HIV.”) and HIV disclosure (e.g., “How did you feel when your mother told you that she is HIV+?” or “How did your child react to the news of your HIV status?”). No specific queries addressed stigma. Mothers’ interviews lasted approximately 75 minutes, and they were compensated for their time with $60 cash. Children’s interviews were approximately 60 minutes, and they received a $30 gift card.

### Measures

#### Demographics

Mothers reported on their age, educational status, employment, household monthly income, race/ethnicity, and marital status. Mothers also reported on their child’s age, gender, and race/ethnicity.

#### Stigma

Mothers reported on their HIV-related stigma as assessed by items from the Stigma Scale (Sayles et al., 2008), which had been adapted for the IMAGE study (Murphy et al., 2016). This study used 15-items from the Stigma Scale to measure three types of stigma: stereotype, social relationships and disclosure. All types of stigma had adequate internal consistency estimates ranging from 0.84–0.90 with the current sample.

#### Mother anxiety

Mothers reported on their anxiety symptoms with the Generalized Anxiety Disorder-7 (GAD-7) questionnaire (Spitzer et al., 2006). It is an 8-item measure that assesses anxiety over the past two weeks. Seven of the prompts evaluated anxiety symptoms with a 4-point Likert scale (1 = not at all; 4 = nearly every day). A final question assessed how difficult the symptoms have been to manage. Higher scores indicate more anxiety symptoms, and Cronbach’s alpha with the current sample was 0.91.

#### Parent-child relationship quality

Children reported on their parental relationship quality with the 20-item Conflict Behavior Questionnaire short form (CBQ; Prinz et al., 1979). The CBQ has been used to assess relationship quality in diverse populations, such as families affected by HIV/AIDS (Armistead et al., 2014). Higher scores indicate better relationship quality, and Cronbach’s alpha with the current sample was 0.89.

#### Child psychosocial adjustment

Mothers reported on their child’s psychosocial adjustment with the Child Behavior Checklist (CBCL; Achenbach, 1991). The CBCL is a 51-item measure of internalizing and externalizing behaviors scored on a 3-point scale (1 = not true; 3 = very true or often true). It has been used in diverse populations (Jones et al., 2002; Tomkins & Wyatt, 2008) and displays good reliability and validity (Achenbach, 1991). With the current sample, the Cronbach’s alpha was 0.92.

### Data Analytic Plan

Mixed methods research maximizes the strengths and protects against the weaknesses present when employing quantitative and qualitative approaches independently to obtain a deeper and more nuanced understanding of phenomena (Creswell, 2015; NIH Office of Behavioral and Social Sciences, 2018). We selected mixed methods given there is sufficient research in the broader field of stigma to allow for hypothesis-driven aims with established measures; and qualitative methods are particularly adept at shedding light on complex or difficult topics, especially with disenfranchised populations (Frost & Ouellette, 2011). Specifically, we used convergent, parallel mixed-methodology by independently collecting and analyzing quantitative and qualitative data without prioritization, and then subsequently mixing findings in the final interpretation (Creswell & Plano-Clark, 2011).

We conducted quantitative analyses using IBM SPSS Statistics, Version 25.0. Initial descriptive statistics provided an overview of the population on study measures. Hierarchical linear regression examined the multivariate association between baseline maternal HIV stigma and child psychosocial adjustment. We entered child age and gender as covariates in Step 1 of the model, parent-child relationship in Step 2, and maternal anxiety in Step 3. Finally, we included HIV stigma in Step 4 as a predictor of child adjustment difficulties.

Trained research assistants transcribed the qualitative interviews. Interviews completed in Spanish were transcribed in Spanish, then translated to English and back-translated for accuracy (Brislin, 1970). Qualitative coding was completed in an iterative process by the collaboration of four coders using NVivo. We employed both, inductive and deductive processes. Specifically, our team of coders independently examined 3 parent and child pairs of transcripts for themes of ‘stigma’ (deductive) and then identified subthemes (inductive) as relevant. The coders then met and discussed the identified subthemes, examined the literature including the Stigma scale (Sayles et al., 2008) for additional clarity and subtheme titles, and ultimately agreed on the titles and definitions for the subthemes. Subsequently, a new set of four parent and four child transcripts were independently coded using the agreed-upon subthemes. The coders then met to discuss any discrepancies in this round of coding and refined the subtheme definitions as necessary. Lastly, the coders independently coded all transcripts. This final round of coding was checked for agreement and any discrepancies were discussed until all coders were in consensus on the categorization of quotes within the subthemes.

## Results

### Quantitative Results

We present descriptive statistics and bivariate correlations for all study variables in Table 1. Overall, mothers reported moderate levels of stereotype stigma (*M* = 20.44, *SD* = 7.70) and disclosure stigma (*M* = 9.91, *SD* = 5.09) and low levels of social relationship stigma (*M* = 6.76, *SD* = 3.57). Mothers reported minimal levels of anxiety (*M* = 2.05, *SD* = 0.87). When reporting their child’s adjustment difficulties, mothers on average indicated problematic behaviors (e.g., argues a lot, lying or cheating, worries) as being “not true” to “somewhat or sometimes true” for their children (*M* = 72.06, *SD* = 13.01). Older children reported better parent-child relationship quality, and their mothers reported that older children had fewer adjustment difficulties than younger children. No gender differences were observed. Better relationship quality was associated with fewer adjustment problems (*r* = −0.28, *p* < 0.001). Mothers’ anxiety was correlated with more HIV-related stigma (*r* = 0.47, *p* < 0.001) and worse child adjustment (*r* = 0.34, *p* < 0.001). HIV stigma was associated with more child adjustment difficulties (*r* = 0.41, *p* < .001). Independent samples t-tests revealed no significant differences across sites (Georgia and California) on HIV stigma, parent-child relationship quality, or maternal anxiety, t(149–178) = −1.33–0.97, p = 0.18 –0.89.

Table 2 presents results of the hierarchical linear regression analysis predicting child adjustment difficulties. In Step 1, child age—but not gender—significantly predicted child adjustment, with older children exhibiting better outcomes (*β* = −0.24, *t* = −3.02, *p* < 0.01). The addition of parent-child relationship quality in Step 2 explained a significantly greater amount of the variance in child adjustment, Δ*R*^*2*^ = 0.08, *F* (3, 143) = 7.42, *p* < 0.001, f^2^ = 0.09. Relationship quality predicted fewer adjustment difficulties (*β* = −0.28, *t* = −3.52, *p* < 0.001). In Step 3, the model including maternal anxiety explained an additional proportion of the variance in child outcomes, Δ*R*^*2*^ = .19, *F* (4, 142) = 16.65, *p* < .001, f^2^ = .23, with maternal anxiety predicting more adjustment problems (*β* = 0.43, *t* = 6.21, *p* < 0.001). Finally, with HIV-related stigma included in Step 4, the model further explained a significantly greater amount of the variance in child adjustment, Δ*R*^*2*^ = 0.04, *F* (5, 141) = 15.97, *p* < 0.001, f^2^ = 0.04. When accounting for the effects of child age and gender, parent-child relationship quality, and maternal anxiety, HIV stigma predicted significantly more child adjustment problems (*β* = 0.23, *t* = 3.05, *p* < 0.01). The final model with all predictors accounted for 36% of the variance in child adjustment, *R*^*2*^ = 0.36, *F*(5, 141) = 15.97, *p* < 0.001, f^2^ = 0.56.

### Qualitative Results

Given the aims of the project, we examined the overall global theme of stigma deductively, and additional themes and subthemes emerged during inductive coding. We found the majority of descriptions about stigma in the parent transcripts; only occasional comments regarding stigma were noted in child transcripts. However, coders agreed that the identified subthemes were consistent across parent and child transcripts, and thus a single set of subthemes were used to code all transcripts. A theme of stereotype stigma emerged, which included subthemes of transmission stigma and misinformation stigma. A second theme was related to disclosure concerns, which included specific subthemes of safe-group and disclosure of mother’s HIV status.

#### Stereotype stigma

Mothers raised concerns about explicit negative judgment about HIV from others or the larger community. For example, one mother stated, “Since I have this illness, they would been looking at me weak.” Another mother stated, “People that’s looking at you, talking about you, ‘nasty, you trash.’ ” A third mother described her conversation with a neighbor who did not know the mother was living with HIV and stated, “I don’t let my children play with some of these children because they momma got AIDS and they do too. They were born with it.” Children also talked about needing to hide HIV, “they tell me how my dad died, so I have to make something up so that way they don’t know.”

#### Transmission stigma

Mothers discussed stigma related to negative perceptions of transmitting HIV. Most of these comments centered on the perception that the mother might have engaged in unprotected and promiscuous sex. A mother raised this issue in a discussion about conversations with her daughter about HIV. “…I explained it to her that not all people see it well. I told her, if people tell you ‘Oh your mom has this…it’s because she was sleeping around with men or other things.’ ” One child also alluded to transmission stigma as they described their reaction to the mother’s disclosure of HIV, “I wasn’t mad with her cause some kids would be. I accepted her because she is my mom and people say it’s bad cause it goes, it passes through or something, like it spreads.”

#### Misinformation stigma

Mothers discussed stigma related to inaccurate or lack of knowledge in others. “A lot of people aren’t educated and think that it is something contagious or by drinking in a cup or using the bathroom.” Another mother commented, “I get frustrated because of, everybody is not that well educated on HIV, so when you open up to someone and let them know of your status, and they don’t know that much about it, they tend to judge you, People are going to think the worst.” Another mother described the persistence of misinformation when discussing her family of origin, “my own family members have talked about me. They don’t want to eat nothing behind me.”

#### Disclosure concerns

As there were specific interview questions around disclosure, mothers discussed this topic in depth. Notably, the responses from the mothers suggested that they experience disclosure-related stigma with most individuals, including their own children.

#### Safe-group

Mothers discussed having a ‘safe group’ of people that their children could turn to for information about HIV. Comments about safe groups were embedded in a larger context of other people—outside of the safe group—having negative views of the mother related to her HIV-status. In response to why she has a safe group, one mother responded, “Well, the fact that people are not well educated.” A child stated, “Once my mom told me not to tell anyone, I haven’t told anyone. So, I don’t have anyone to talk to.”

#### Disclosure of mother’s HIV status

Mothers raised issues about stigma related to the disclosure of their HIV + status to their children and others:

“I made sure to tell him no one knew about it, I told him, ‘Don’t talk to anyone about it, keep silent.’ And imagine, if they ever were to find out? It’s for that reason that people start taking away this and that and people start ‘oh, no get away.’ So it’s better to keep it that way and then he started asking me ‘Mom does granny know? Do my aunts know?’ And I tell him ‘No one knows, you keep silent, don’t say anything, stay quiet.’ ”

In particular, mothers talked about the importance of being the ones to disclose to their own children due to potential negative or inaccurate information that might come from other people. For example, one mother stated, “It’s better for you to tell her because sometimes outside people don’t know how it is and if your daughter finds out from other people, people have a bad connotation of the disease.” This MLH also referred to the importance of disclosing having a stigmatized illness in terms of maintaining a good relationship with her child, “I would also say ‘if your daughter looks at you like this and sees that you are strong that you are not like those people then I would say do it when she’s, when, right now when you still have trust.” Similarly, another mother talked about disclosure to her child as a mechanism for combatting stigma:

“Me being so public about my status, understanding that other people could come to him, and understanding what type of confidence he would need to be able to umm, combat any stigma, because I understood that me sharing my status, and when I say publicly I mean like, nationally and internationally like, this is in campaigns in stuff and if you see mommy on a TV commercial or billboard you know then how are you going to respond to that.”

## Discussion

HIV is a persistent public health concern, and its stigma continues to plague those living with HIV and their families (Lipira et al., 2018). The average response of mothers in the current study indicated that they perceived stigma as illustrated by their responses of at least “some of the time” to measure items including, “People assume I have done something bad to get HIV” and “People avoid me because I have HIV.” Further, whereas mothers’ quantitative reports indicated moderate stereotype and disclosure-related stigma, they reported lower levels of social relationship stigma (e.g., “I feel abandoned by my family members because I have HIV” and “People I am close to are afraid they will catch HIV from me).” The qualitative interviews, though only conducted with a subset of quantitative participants, magnified stigma-related concerns, including those tied to social relationships. Specifically, though the qualitative interviews were not constructed to solicit information about stigma, we deduced from theory and qualitative review of the interviews that a majority of MLH participants still mentioned aspects of stigma in their responses (Berger et al., 2001; Earnshaw & Chaudoir, 2009; Nyblade, 2006; Sayles et al., 2008). For example, MLH raised concerns about how others perceive them, including family members, neighbors, and society as a whole. They also described delaying disclosure to their children or other family members for fear that HIV-stigma would compromise how the MLH or her family are perceived by others. Striking is the similarity in quotes about the disclosure of their HIV status from MLH participating in a study published by Murphy et al., 2002 (e.g., “I told him not to tell anybody.”) and MLH in the current study, conducted over 15 years later (e.g., “I made sure to tell him no one knew about it, I told him, ‘Don’t talk to anyone about it, keep silent.’ ”).

HIV compromises maternal psychological functioning (e.g., increased symptoms of anxiety) and aspects of parenting (e.g., the parent-child relationship), which can lead to problems in child adjustment. The quantitative results of the current study indicate that children appear to be impacted, not only by these well documented HIV-related risks to families, but also by their mothers’ experience of stigma, which is consistent with some (Gamarel et al., 2017; Murphy et al., 2006), though not all (Bauman et al., 2002) of the extant literature. Our study’s findings are also anticipated by Pachankis’ cognitive-behavioral-affective model for the concealment of stigma. Particularly notable is the statistically significant association between child functioning and maternal stigma after controlling for both mother-child relationship quality and mothers’ symptoms of anxiety.

In contrast, the qualitative data offer only limited support for a connection between HIV stigma and child functioning. Only a few children mentioned stigma specifically or described situations clearly driven by stigma. Indeed, based on MLH qualitative interviews, we were somewhat surprised by the lack of concern that child qualitative participants expressed about stigma. No MLH or children described a clear link between stigma and the child’s functioning, though MLH did express that concerns about stigma delayed disclosure to children, which may, in turn, impact child functioning (Qiao et al., 2013). The inconsistency in quantitative and qualitative findings is likely explained in part by the fact that stigma was not a focus of the qualitative interviews and only emerged organically during participants’ responses to interviewer prompts about other issues.

Results must be considered in light of study limitations. The quantitative data were collected only from families in which disclosure of the mothers’ HIV status had not occurred, whereas qualitative data were collected from a subsample in which mothers had disclosed subsequent to receiving the TRACK intervention. Generalizability to families disclosing outside of a research study should be carefully considered (Goodrum et al., 2021). In particular, based on the disclosure model of TRACK, we would hypothesize that MLH who independently disclose experience less stigma and anxiety than those who do not disclose or disclose following receipt of a disclosure intervention (Derlega et al., 2004). Another limitation is that the data are self-reported and cross-sectional; thus, the directionality of the relationships cannot be confirmed. Further, because children had not been told about their mothers’ HIV at the point of quantitative data collection, children’s perception of HIV stigma could not be assessed. Future work could examine the potential additive effects of MLH’s and children’s perception of stigma on child functioning. Finally, the qualitative interviewers did not provide specific prompts to elicit stigma concerns. Thus, though most MLH talked about stigma, in the absence of prompts, the researchers may have missed the opportunity to gather stigma-related concerns from children.

In this study, utilizing mixed methods, we found stigma to be an important consideration in families with an MLH, which calls for expanded efforts to combat HIV stigma. Children whose mothers are living with HIV already bear the burden of maternal illness, often along with residence in under resourced communities. Comprehensive interventions can mitigate impact of these risk factors. Based on the study’s qualitative interviews, it is of concern that so much of their content mimics findings from one of the earliest studies on children carrying the secret of their mothers’ serostatus (Murphy et al., 2002). Despite the fact that HIV is now considered a chronic illness and there has been a widespread and intensive effort to educate the public about HIV transmission, misconceptions that contribute to enacted stigma persist as indicated by the mother whose neighbor did not let her children play with some children because their mother is living with HIV. Additionally, the association between MLH’s perceived stigma and child outcomes, after accounting for maternal anxiety and parent-child relationship quality, appears to confirm something postulated by Murphy et al. (2006): Support for children who have not been told of their mothers’ HIV status may be just as crucial as for those aware of their mothers’ serostatus.

## Figures and Tables

**Table 1:** Descriptive statistics and bivariate correlations among all study variables

Variable	*M*	*SD*	1	2	3	4	5	6
1. Child Age	9.67	2.49	.					
2. Child Gender	50.3%	Female	−0.02	.				
3. Parent-Child Relationship Quality	36.52	3.92	0.15*	−0.04	***0.86***			
4. Maternal Anxiety	2.05	0.87	0.04	0.04	0.01	***0.91***		
5. Maternal HIV Stigma	37.28	14.17	−0.02	−0.09	−0.12	0.47***	***0.90***	
6. Child Adjustment Difficulties	72.06	13.01	−0.20**	−0.03	−0.28***	0.34***	0.41***	***0.92***

Scale reliabilities (Cronbach’s alpha) are shown in ***bolded italics*** on the diagonal

* *p* < 0.05;

** *p* < 0.01;

*** *p* < 0.001

**Table 2:** Hierarchical regression analysis predicting child psychosocial adjustment

Predictor	β	*t*	*F*	Δ*R*^2^
Step 1			4.57*	
Child Age	−0.24	−3.02**		
Child Gender	0.02	0.26		
Step 2			7.42***	0.08**
Child Age	−0.19	−2.32*		
Child Gender	0.01	0.137		
Parent-Child Relationship Quality	−0.30	−4.20***		
Step 3			16.65***	0.19***
Child Age	−0.19	−2.71**		
Child Gender	−0.05	−0.69		
Parent-Child Relationship Quality	−0.30	−4.20***		
Mothers’ anxiety	0.43	6.21***		
Step 4			15.97***	0.04**
Child Age	−0.19	−2.76**		
Child Gender	−0.05	−0.80		
Parent-Child Relationship Quality	−0.27	−3.87***		
Mothers’ Anxiety	0.33	4 27***		
Mothers’ HIV Stigma	0.23	3.05**		

* *p* < 0.05;

** *p* < 0.01;

*** *p* < 0.001

## Highlights

- Maternal HIV stigma is significantly associated with child adjustment difficulties.
- HIV stigma persists as a significant concern for families affected by maternal HIV.
- Qualitative reports of HIV stigma are consistent with ones from over 15 years ago.
